# Shedding light on the role of keratinocyte-derived extracellular vesicles on skin-homing cells

**DOI:** 10.1186/s13287-020-01929-8

**Published:** 2020-09-29

**Authors:** Golara Nasiri, Negar Azarpira, Aliakbar Alizadeh, Sanaz Goshtasbi, Lobat Tayebi

**Affiliations:** 1 Department of Tissue Engineering and Cell Therapy, School of Advanced Technologies in Medicine, Shiraz University of Medical Sciences, Shiraz, Iran; 2 Transplant Research Center, Shiraz University of Medical Sciences, Khalili Street, Shiraz, 7193711351 Iran; 3 Marquette University School of Dentistry, Milwaukee, WI 53233 USA

**Keywords:** Extracellular vesicles, Keratinocytes, Fibroblasts, Melanocytes, Immune cells, Skin

## Abstract

Extracellular vesicles (EVs) are secretory lipid membranes with the ability to regulate cellular functions by exchanging biological components between different cells. Resident skin cells such as keratinocytes, fibroblasts, melanocytes, and inflammatory cells can secrete different types of EVs depending on their biological state. These vesicles can influence the physiological properties and pathological processes of skin, such as pigmentation, cutaneous immunity, and wound healing. Since keratinocytes constitute the majority of skin cells, secreted EVs from these cells may alter the pathophysiological behavior of other skin cells. This paper reviews the contents of keratinocyte-derived EVs and their impact on fibroblasts, melanocytes, and immune cells to provide an insight for better understanding of the pathophysiological mechanisms of skin disorders and their use in related therapeutic approaches.

## Background

The skin is a multilayer tissue organization that covers the whole body and thus is known as the largest organ. Protecting the body against microorganisms and hazardous materials and regulating the rate of dehydration in response to temperature, as well as sense of pressure and pain, are the most important functions of the skin. Various factors—such as environmental stimuli and hormones—influence the structural stability and function of the skin [1].

The structure of the skin is layered, and each layer is made up of various cell types and biomolecules [2]. The first layer, epidermis, provides an environmental barrier to pathogens and controls water loss from the body. The epidermis is composed of diverse cells including keratinocytes, melanocytes, Langerhans cells, Merkel cells, inflammatory cells, and stem cells [3]. The second layer, dermis, primarily contains nerve endings, blood vessels, and cells such as fibroblasts, macrophages, and adipocytes. The last layer, hypodermis, consists mainly of fat and blood vessels and contains the same cells as the dermis.

Among different skin cells, keratinocytes, fibroblasts, and melanocytes have close communication with one another. Keratinocytes establish a tight stratified layer by a highly regulated differentiation process of progenitor cells [4]. Fibroblasts synthesize the extracellular matrix proteins and interfere in inflammatory responses [5]. The intercommunication between keratinocytes and fibroblasts is essential during the mechanism of wound healing. Furthermore, basal keratinocytes are recipient cells for melanin pigment secreted by melanocytes.

Intercellular communication is an important issue for survival of life in multicellular organisms. The extracellular matrix (ECM), which is known as cellular microenvironment, plays a critical role in intercellular communications. ECM is an extensive three-dimensional network made from a variety of proteins, proteoglycans/glycosaminoglycans, and glycoproteins. Cells produce ECM in response to growth factors, cytokines, and mechanical signals via their surface receptors [6].

Communication between cells is also mediated by the EVs. EVs are small fragments detached from the plasma membrane of cells that are released into ECM. It has been demonstrated that EVs can communicate between different cells, since they contain cellular biological components that may affect cell signaling pathways. Regarding skin tissue, different skin cells such as keratinocytes, fibroblasts, melanocytes, and inflammatory cells also communicate with one another through EVs that is mediated by ECM. Regarding skin tissue, different skin cells such as keratinocytes, fibroblasts, melanocytes, and inflammatory cells also communicate with one another through EVs that is mediated by ECM.

The nature of EVs as biological carriers has potential for exploitation in different skin therapy purposes including repair, regeneration, and rejuvenation [7]. However, to make these therapeutic approaches accessible to patients in the future, it is necessary to develop good manufacturing practice (GMP) as a standard protocol to ensure the quality of EVs used [8, 9]. In this regard, this review focuses mostly on the keratinocyte-derived EVs and their impact on biological behavior of other skin cells.

### Extracellular vesicles

Cells generally communicate using secreted factors or membrane vesicles, commonly known as EVs. EVs are phospholipid bilayer membranes that are classified into exosomes (EXs), microvesicles (MVs), and apoptotic bodies (ApoBDs) based on their different biological characteristics (Table 1) [10–15].

**Table 1:** Comparison between the characteristics of extracellular vesicles

Type	Size	Content	Surface markers	Biogenesis origin	Isolation method
**Exosomes**	40–100 nm	mRNA, miRNA, and other non-coding RNAs; lipids (cholesterol, ceramide, sphingomyelin); cytoplasmic and membrane proteins including receptors and MHC molecules, lower amount of DNA	Tetraspanins (CD63, CD9, CD81), Alix, Hsp60, Hsp70, Hsp90, clathrin, annexins, ESCRT components (PDCD6IP and TSG101), flotillin	Formation of early endosome/formation of late endosome/formation of MVB/fusion with cell membrane and exocytosis	Immunoprecipitation (ExoQuick®), ultracentrifugation, (100,000–200,000*g*), ultracentrifugation with density gradient
**Microvesicles**	50–1000 nm	mRNA, miRNA, non-coding RNAs, cytoplasmic proteins, and membrane proteins, including receptors, Integrins, selectins, MMPs, phosphatidylserine, cholesterol, sphingomyelin, and ceramide	Integrins, selectins, MMPs, phosphatidylserine, CD40, ARF6, VAMP3	Cell membrane zeiosis	Ultracentrifugation (10,000–60,000*g*)
**Apoptotic bodies**	800–5000 nm	Cell organelles, nuclear fractions including DNA, rRNA, mRNA	Phosphatidylserine	Programmed cell death-mediated zeiosis and cell fragmentation	FACS and differential centrifugation

EXs and MVs are released by living cells. They are produced by most, but not all, cell types and similarly contain genetic material such as mRNA, miRNA, and even lower amount of DNA, along with numerous proteins [16]. EXs are 40–100 nm in size and constitute a homogeneous group of EVs [17], while MVs are more heterogeneous (50–1000 nm). Alix, HSP70, and the tetraspanins—such as CD9 and CD63—are some of the surface markers of EXs, whereas integrins, selectins, and CD40 are found on the surface of MVs. The biogenesis mechanism of EXs is not fully understood; however, it is commonly accepted that EXs are formed through the endocytosis-exocytosis pathway. Generally, the formation process begins when intracellular fluid is internalized by different endocytic pathways to form early endosomes. During the next step, early endosomes are developed into late endosomes, which mature subsequently to become multi-vesicular bodies (MVBs). Lastly, MVBs are fused with the cell membrane and exocytosis into the extracellular environment, known as EXs. In contrast to EXs, MVs originate from direct budding of the cell membrane, which is dependent on calpain (a calcium-dependent protein), cytoskeleton reorganization, and intracellular calcium concentration. Calcium ions are responsible for phospholipid redistribution of cell membrane that leads to the formation of MVs.

In comparison to EXs and MVs, ApoBDs are the largest EVs (800–5000 nm) generated from many apoptotic cell types that mainly contain organelles and nuclear components. ApoBDs express the same surface markers as their cells of origin, which might be used to discriminate cell type-unique ApoBDs. However, phosphatidylserine is the only common marker of ApoBDs which has been identified so far [18]. ApoBDs are released into the ECM through several stages, beginning with chromatin condensation and nuclear splitting, then budding of the cell membrane followed by proteomic degradation, and finally disintegration of the cellular content into distinct membrane enclosed vesicles, termed as ApoBDs.

### The characteristic of keratinocyte-derived EVs

Than et al. utilized a differential centrifugation method to isolate EVs from spontaneously immortalized human keratinocytes, HaCaT (human adult high calcium low temperature [19]) cells, and primary human keratinocyte cell cultures (PHKCs), then examined their contents [20]. Heat shock protein 70 (HSP70), tumor susceptibility gene 101 (TSG101), argonaute 2 (AGO2), CD81, CD63, and CD9 were expressed by the three different EVs populations. Membrane proteins (integrin alpha 6 (ITGA6), CD9, CD63) and cytoplasmic proteins (heat shock 70 kDa protein 5 (HSPA5), eukaryotic translation elongation factor 1 alpha 1 (eEF1A1), syndecan binding protein (SDCBP)) were common proteins corresponding to HaCaT- and PHKCs-derived EVs. These proteins are necessary for skin development and repair (ITGA6), cell adhesion (CD9), or cell extension and migration (CD9, SDCBP) [21–25]. ECM related proteins—including laminins, integrins, collagens, tenascins, thrombospondins, and syndecans, along with some growth factors, cytokines, integrins, metalloproteinases, stratifins, and cadherins—were found in HaCaT- and PHKCs-derived EVs [20].

Additionally, the characterization of keratinocyte EVs confirmed the existence of other proteins like transforming growth factor beta (TGF-β), epidermal growth factor (EGF), involucrin, kallikrein 7 (KLK7), jagged 1 (JAG1), plasminogen activator inhibitor 1 (PAI-1), matrix metalloproteinase-1, -3, -8, and -9 (MMP-1, MMP-3, MMP-8, MMP-9), mitogen-activated protein kinase 3 (MAPK3), and lactate dehydrogenase (LDH) [26]. TGF-β regulates skin homeostasis and regeneration by stimulating the fibroblasts [27] to express collagen and other extracellular proteins, while EGF, involucrin, KLK7, and MAPK3 are potentially involved in keratinocyte differentiation. In addition, data has suggested that JAG1 is associated with the enhancement of fibroblast growth factor in vitro, while PAI-1 can play role in keratinocytes adhesion and migration during wound healing [28]. Matrix metalloproteinases (MMPs) are crucial enzymes for wound re-epithelialization, as they contribute in extracellular matrix degradation and deposition during tissue injuries [29]. Lastly, LDH can induce endothelial cell growth, proliferation, and migration by stimulating vascular endothelial growth factor (VEGF) production [30].

The biological status of keratinocytes can also determine the contents of their secreted EVs. For instance, the activated migrating keratinocytes secrete EVs contain cathepsin B, which interferes in intracellular proteolysis during wound healing [31]. Furthermore, different isoforms of 14-3-3 protein have been identified in exosomes from differentiated and undifferentiated keratinocytes [26]. All seven 14-3-3 protein isoforms (β, σ, η, ε, τ, ζ, and γ) exist in the exosomal cargos of differentiated keratinocytes; however, exosomes of undifferentiated keratinocytes only contain β, η, ζ, and γ isoforms. Each of these isoforms is involved in different biological functions. For example, it has been demonstrated that 14-3-3σ can regulate protein synthesis and epithelial cell growth by binding to keratin 17 (KRT17). This isoform is also known as stratifin, which acts as a collagenase stimulator factor in dermal fibroblasts [32]. 14-3-3γ and β proteins act as protein kinase C (PKC) inhibitors. Meanwhile, 14-3-3ε takes part in signal transduction and cellular division and, along with 14-3-3ζ, both are involved in the insulin sensitivity modulation. Lastly, 14-3-3τ isoform is known as 14-3-3 protein T cell and a protein kinase regulator [26].

Regarding microRNAs, the let-7 family—including hsa-miR 22, hsa-miR 27b, and hsa-miR 21—were among the common miRNAs. The let-7 miRNAs regulate various cellular activities, such as proliferation, differentiation, and apoptosis [33]. hsa-miR 22, hsa-miR 27b, and hsa-miR 21 regulate cell proliferation and apoptosis, fibroblast activation, and keratinocyte migration, respectively. Additionally, miRNA-203 and miRNA-205 were discovered in large amounts [20], which could affect the wound closure process by regulating keratinocyte proliferation and migration [34, 35]. Some of the keratinocyte-derived components and their relevant functions are presented in Table 2.

**Table 2:** Some of the keratinocyte-derived extracellular vesicle components and their biological functions

**CD9**	Exosome biogenesis pathways, cell adhesion
**CD63**	Exosome biogenesis pathways
**hsa-miR 22**	Regulation of cell proliferation and apoptosis
**hsa-miR 27b**	Fibroblast activation
**hsa-miR 21**	Keratinocyte migration, fibroblast migration and differentiation, fibroblast-mediated angiogenesis and pro-inflammatory response
**miRNA-203, miRNA-205**	Regulation of keratinocyte proliferation and migration
**Cathepsin B**	Intracellular proteolysis
**Hsp70**	Cytoprotection
**Annexin II**	Biogenesis of the EVs
**Mac-2BP**	Cell adhesion and host defense
**TGF-β**	Stimulation of fibroblast to express collagen
**EGF, involucrin, KLK7, MAPK3**	Keratinocyte differentiation
**Jagged-1**	Regulation of fibroblasts growth factor
**PAI-1**	Keratinocytes adhesion and migration
**MMPs**	Extracellular matrix degradation
**LDH**	Stimulation of VEGF production
**14-3-3σ**	Stimulation of fibroblast to express collagen
**14-3-3 γ/β**	PKC inhibition
**14-3-3ε**	Signal transduction and cell division
**14-3-3ζ**	Regulation of insulin sensitivity
**14-3-3τ**	Regulation of protein kinase
**ITG**	Communication between keratinocytes and fibroblasts
**Hsp90α**	Pro-motility factor for the migration of keratinocytes, fibroblasts and endothelial cells

### Cross-talk between keratinocytes and fibroblasts through EVs

Keratinocyte-fibroblast interactions occur via soluble mediators or EVs that contain signaling molecules. These epidermal-dermal communications are necessary not only for the maintenance of skin homeostasis, but also for the process of wound healing.

The communication between keratinocytes and fibroblasts can be mediated by integrins (ITG), which are heterodimeric cell adhesion receptors that transmit biochemical and biomechanical signals between the cell and its surrounding matrix. The signal transduction is necessary for different cellular activities such as differentiation, migration, expression of specific genes, or apoptosis. EVs containing ITGα1, ITGα2, ITGα3, ITGα6, ITGβ1, ITGβ3, and integrin-specific accessory molecules released by primary human epidermal keratinocytes have been identified in the ECM of epidermis layer [36], and therefore, it is hypothesized that EVs from epidermal keratinocytes may deliver their integrins to fibroblasts during healing processes.

It has been demonstrated that EVs can affect gene expression in fibroblasts. For example, Huang et al. indicated the presence of MV-like vesicles during active keratinocyte migration and early stages of granulation tissue organization in human wounded skin [37]. They found that the addition of keratinocyte-derived MVs to fibroblast culture medium could affect the expression of MMP-1 and -3, IL-6 and -8, and also the genes associated with TGFβ signaling pathway, like cellular communication network factor-2 and -3 (CCN2, CCN3), elastin microfibrillar interface-located protein 3 (Emilin-3), collagen triple helix repeat containing 1 (CTHRC1), NGFI-A binding protein 1 (NAB1), and thrombospondin 1 (THBS1).

Experimental studies using PD184352 (an inhibitor for ERK1/2 signaling pathway) indicated that the expression of MMP1, MMP3, IL6, and IL8 was mediated by ERK signaling, which caused the significant overexpression of these genes [37]. Generally, during the wound healing process, MMP production by fibroblasts influences ECM remodeling and provides keratinocyte participation in tissue remodeling. Furthermore, both IL-6 and IL-8 contribute to wound healing by stimulating macrophage infiltration, keratinocyte migration, and angiogenesis [38]. As a result, both fibroblasts and keratinocytes have mutual effect on each other in order to improve the ECM remodeling for wound healing**.**

In the vicinity of keratinocyte-MVs, the expression of CCN2 in fibroblasts increased while CCN3 decreased [37]. CCN2 and CCN3 are downstream genes (mediator/target) of TGF-β signaling [39]. In human skin fibroblasts, CCN2 exhibits activities such as matrix production, cell migration and promotion of pro-angiogenic genes expression [40–42], while CCN3 blocks CCN2 and decreases matrix production [43].

THBS1, Emilin-3, CTHRC1, and NAB1—which are involved in TGF-β signaling modulation—were upregulated by the keratinocyte-MVs [37]. THBS1 is essential for initiating TGF-β signaling in latent TGF-β [44], whereas Emilin-3 and CTHRC1 serve as inhibitors of the TGF-β signaling pathway [45–47]. Finally, NAB1 acts as a corepressor of the early growth response 1 (Egr-1), which is required for abrogation of TGF-β-stimulated fibrotic responses [48, 49]. In general, it can be concluded that keratinocyte-MVs mediate expression of both positive and negative regulators of TGF-β signaling pathway, which leads selective regulation of ECM protein genes.

Besides affecting gene expression, keratinocyte-MVs may also influence on the phenotype of fibroblasts. In the same study, Huang et al. found that keratinocyte-MVs have contradictory effect on fibroblast differentiation to myofibroblasts by reducing expression of alpha-smooth muscle actin (α-SMA) and cadherin-2 [37]. During natural healing process, fibroblast differentiation is largely regulated by TGF-β pathway and during this phenotype alteration, α-SMA expression is induced and cadherin-2 is changed to cadherin-11 [50]. Therefore, keratinocyte-MVs appear to have dual role in regulation of fibroblast differentiation by preparing them towards phenotype change (cadherin-2 downregulation), but obstructing the full differentiation (α-SMA reduction).

Likewise, Huang et al. investigated the effect of keratinocyte-MVs on the fibroblast genes related to angiogenesis (vascular endothelial growth factor-A (VEGF-A), fibroblast growth factor 2 (FGF-2), and C-X-C motif chemokine ligand 12 (CXCL12)), and they revealed that only expression of FGF2 was dose-dependently upregulated by the keratinocyte-MVs [37].

Importantly, activation of ERK1/2, JNK, Smad, and p38 signaling pathways occurred under the influence of keratinocyte-derived MVs [37]. These signaling pathways resulted in fibroblast migration and promoted the endothelial tube formation.

Recently, miR-21 has been found in the contents of keratinocyte-derived MVs. The involvement of miR-21 in different cellular activities—such as proliferation, differentiation, migration, apoptosis, and epithelial to mesenchymal transition (EMT)—is well established [51]. Qian et al. found that miR-21-MVs derived from keratinocytes are implicated in fibroblast migration and differentiation, fibroblast-mediated angiogenesis, and pro-inflammatory response [52]. The results also confirmed the contribution of miR-21-MVs on MMP-1, MMP-3, IL-6, and IL-8 overexpression in fibroblasts in which phosphatase and tensin homolog (PTEN), regulator of IL-6 and IL-8 expression, was found to be the target of miRNA-21.

In addition to keratinocyte-derived EVs, the interaction between fibroblast-derived EVs and keratinocytes has also been revealed. For instance, Terlecki-Zaniewicz and colleagues compared the effect of senescent and quiescent fibroblast-derived EVs on the dynamic of scratch closure assay in an in vitro 2D culture model [53]. They identified miR-23a-3p in the contents of fibroblast-derived EVs, which has a crucial role in cellular senescence [54] and skin aging. The results showed that temporary exposure of keratinocytes to senescent cell-derived EVs could increase the number of keratinocytes in the cell free area, in comparison to those that were subjected to EVs from quiescent fibroblasts. However, prolonged incubation impaired the keratinocyte differentiation in vitro. This was in line with in vivo evidence showing increased wound healing [55] as a result of temporary presence of senescent cells.

### Cross-talk between keratinocytes and melanocytes through EVs

Melanocytes are specialized cells that are situated in the basal layer of epidermis and produce melanin. The most important role of melanin is the prevention of UV-induced DNA damage in human keratinocytes, achieved by filtering harmful UV radiation. Melanocytes provide specific organelles, termed melanosomes, in which melanin pigment is produced and deposited. In fact, melanosomes are shedding vesicles that are taken by the microvilli of keratinocytes [56]. In other words, melanosomes can be considered as a type of EV, which enables the communication between melanocytes and keratinocytes that result in skin pigmentation.

Melanocytes can interact with keratinocytes through filopodia. Filopodia, which are correlated with the production of pigment globules and originated from the melanocyte dendrites, were initially discovered to serve as conduits for melanosome transmission to the keratinocytes [57]. Ando et al. revealed the mechanism of transferring melanosomes from melanocytes to keratinocytes [56]. Pigment globules containing melanosomes bud off from all areas of melanocyte dendrites, are secreted into the ECM, phagocytosed by keratinocytes, and then dispersed in the keratinocyte cytosol followed by gradual degradation of the membrane surrounding the melanosome. However, whether a single transfer mechanism or other multiple mechanisms are involved in the melanosome transfer remained unclear.

Ultraviolet (UV) irradiation causes skin pigmentation, which depends on the intercellular interchange of melanin between melanocytes and keratinocytes. When melanocytes are exposed to ultraviolet A (UVA), EVs shedding from plasma membrane occurs and then these EVs are preferably captured by keratinocytes [58]. Similarly, ultraviolet B (UVB) can affect intercellular communication between melanocytes and keratinocytes through EXs, shedding from keratinocytes [59].

The secreted exosomes from keratinocytes can promote melanin synthesis by improving the expression and function of melanosomal proteins [59]. However, the function of these exosomes is distinct in different skin phototypes and can be adjusted by UVB. According to results conducted by Lo Cicero et al., the expression of melanocyte proteins—including tyrosinase (TYR), microphthalmia-associated transcription factor (MITF), and Rab27a—were increased when melanocytes were exposed to miRNA-derived exosomes obtained from UVB treated Caucasian and Black keratinocytes, in comparison to non-treated Caucasian keratinocytes [59]. hsa-miRNA-3196 and hsa-miRNA-203 are specific microRNAs that correlate with this pathway. In fact, hsa-miRNA-3196 upregulated the expression of MITF and Rab27a, while hsa-miRNA-203 caused upregulation of TYR and Rab27a [59]. On the other hand, the reduction of MITF levels in melanocyte cells in the presence of keratinocyte exosome-derived miR-675 has also been demonstrated [60].

In another study, Liu et al. found that keratinocyte-derived EXs carry miR-330-5p-targeting TYR, which could suppress melanocyte pigmentation by inducing a significant decrease in the production of melanin and expression of TYR. Additionally, overexpressed miR-330-5p in melanocytes also proved the inhibitory effect of miR-330-5p on pigmentation [61].

Since EVs have potential to deliver therapeutic agents, such as DNA, mRNA, miRNA, or peptide sequences, exclusive bioengineering of EXs [62, 63] containing genes like miR-330-5p, miR-675, miRNA -3196, or miRNA-203 could offer a therapeutic approach for hypo- and hyperpigmentation disorders by regulating the balance of expression of melanocyte proteins.

### Cross-talk between keratinocytes and immune cells through EVs

Keratinocytes are the first skin cells that encounter environmental allergens; therefore, they play a critical role in skin immunity by organizing the physical barrier. Furthermore, previous publications revealed a high level of allergen uptake by keratinocytes in inflammatory conditions like chronic atopic eczema [64]. Keratinocytes can operate as modulators for the migration of inflammatory cells, keratinocyte proliferation, or differentiation, as well as the induction of other cytokines through cytokine secretion [65], which can be mediated by EVs. In addition, they produce and secrete various antimicrobial peptides (AMPs) such as cathelicidin LL-37, a family of AMPs implicated in the pathogenesis of inflammatory skin disease, like psoriasis [66]. It is noteworthy that keratinocytes can stimulate rapid reaction in antigen specific memory CD4+ and CD8+ T cells by processing endogenous and exogenous antigens [67]. It has been proven that epidermal keratinocytes intrinsically express MHC I and display instigated expression of MHC II under inflammatory conditions [68, 69].

As previously mentioned, keratinocytes release extracellular vesicles. Secreted exosomes from keratinocytes function as intercellular transmitters and immune modulators through interaction with antigen-presenting cells (APCs). For instance, keratinocyte-EXs are found to be internalized by dendritic cells (DCs) in vitro.

In a study conducted by Yin et al., exosome transfer from the mouse progenitor epidermal keratinocyte (MPEK) cell line to bone marrow-derived dendritic cells (BMDCs) was analyzed under both steady state and inflammatory conditions (e.g., in the presence of +/IFNc) [70]. Interestingly*,* BMDCs readily took up these exosomes in vitro and matured, as they overexpressed CD40 and increased the production of IL-6, IL-10, and IL-12. The investigation of antigen-specific information transportation via exosomes confirmed that keratinocytes picked up antigens and delivered them to their exosomes [70]. However, MPEK-derived exosomes containing antigens failed to activate antigen-specific T cells via BMDCs.

It is well determined that exosomes from mature DCs are effective immune activators [71], while immature DCs have been proven to be immunosuppressive. The major reason for this difference is due to their content of proteins involved in immune modulation [72]. Therefore, since keratinocytes are non-professional APCs, their exosome contents are more similar to those of immature rather than that of mature DCs, thus indicating an anti-inflammatory activity for keratinocyte-EXs. Altogether, this finding suggests that keratinocytes are capable of directing nonspecific immune responses but do not evoke specific immune system. However, the factors that mediate these changes remained unknown.

It is proposed that under inflammatory conditions or in the presence of superantigens, T cell activity is stimulated by keratinocytes. For example, Staphylococcal enterotoxin B (SEB) is such a superantigen produced by *Staphylococcus aureus* bacterium. SEB binds to MHC II and, with less affinity, to the T cell antigen receptors without MHC molecules, causing severe stimulation of the immune system and provoking acute pathological effects [73].

In this regard, Cai et al. analyzed the effects of pretreated HaCaT cells (with interferon γ and SEB) on the function of rested T cells [74]. The HaCaT cells secreted exosomes with CD63 and TSG101 markers that promoted SEB/IFNγ-associated expansion of resting CD4+ and CD8+ T cells in vitro, even though the mechanism of interaction between exosomes and T cells was not specified. However, earlier studies proposed that individual exosomes may bind to the specific receptors on target cell and be internalized by endocytosis or by plasma membrane fusion [75, 76]. For example, it has been demonstrated that T cells can recruit released exosomes from DCs, via their LFA-1 receptors [76].

Regarding superantigen-mediated inflammation, it should be noted that the SEB superantigen is implicated in the pathogenesis of psoriasis diseases in more than 50% of cases [77]. Psoriasis is a chronic inflammatory disorder that severely affects the skin and nails. As keratinocytes are the most predominant cell type in epidermis, they are also the main cells with which *Staphylococcus aureus* contacts. Dysregulated keratinocytes along with different kinds of penetrated immune cells orchestrate an abnormal immune response. For example, neutrophils can amplify inflammatory processes by producing neutrophil extracellular traps (NETs) [78], which recently were found to be associated with psoriasis progression. It has been reported that NETs may induce antimicrobial peptide human beta-defensin-2 (HBD-2) expression in psoriatic keratinocytes [79] and the induction of T helper 17 cells from peripheral blood mononuclear cells (PBMCs) [80].

Relatedly, Jiang et al. investigated the release and function of psoriatic keratinocyte-EXs and focused on the communication between keratinocytes and neutrophils [81]. In order to simulate psoriasis condition, keratinocytes were treated with psoriatic cytokine cocktail and then released exosomes from both healthy and psoriatic keratinocytes were characterized. The results determined that psoriatic keratinocyte exosomes could significantly promote the formation of NETs and subsequent expressions of IL-6, IL-8, and tumor necrosis factor-alpha (TNF-α) in neutrophils, which have been reported to play a critical role in psoriasis [82]. For instance, high level IL-6 in wounded tissue has been reported as required for the dampened regulatory T cell activity detected in psoriasis patients [83]. IL-8 is able to attract more neutrophils to the lesion site [82] and TNF-α can be found in many inflammatory diseases including psoriasis [84]. However, a specific cargo in psoriatic keratinocyte-EXs that might be responsible for stimulating the activation of neutrophils was not identified [81]. On the other hand, NF-kB and p38 MAPK signaling pathways were activated in neutrophils, induced by cytokine-treated keratinocyte-EXs and were responsible for the expressions of mentioned proinflammatory factors.

By considering this hypothesis that keratinocyte-EXs are associated with superantigen-related inflammatory diseases, the design of exosomes inhibitor mechanism may offer a potential therapeutic approach through the reduction of immune responses [74].

### The effect of EVs on the wound healing process

**Fig. 1:**
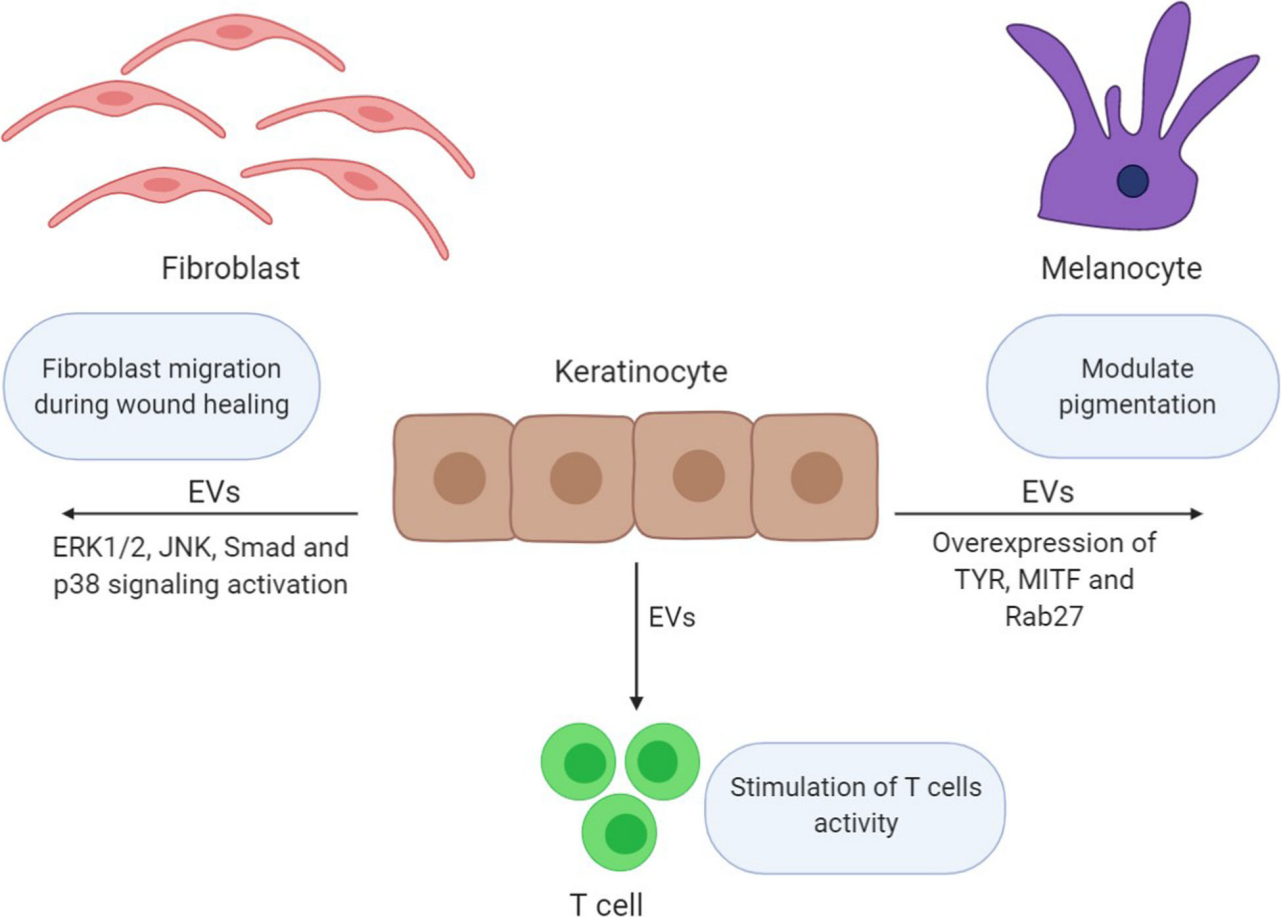
The relationship between keratinocyte-derived extracellular vesicles with wound healing process

Intercellular communications are important for maintaining tissue hemostasis during injury, and the role of EVs is significant in establishing these communications, as they are able to influence cell proliferation, migration, and angiogenesis. Secreted EVs from keratinocytes can motivate fibroblast migration by activating specific signaling pathways and can improve skin diseases by regulating pigmentation in melanocytes and controlled stimulation of immune cells activity (Fig. 1).

Following skin injury, released transforming growth factor-alpha (TGFα) from epidermal cells stimulates secretion of the exosomes containing heat shock protein 90-alpha (HSP90α) proteins from keratinocytes [85]. HSP90α is a common pro-motility factor for the migration of main human skin cells during wound healing process through binding to low-density lipoprotein receptor-related protein 1 (LRP-1) on the surface of keratinocytes, dermal fibroblasts, and dermal microvascular endothelial cells [86]. Extracellular HSP90a acts as a ligand that binds to subdomain II among four external subdomains of LRP-1, to trigger cross membrane signal transduction inside the cell. The signal crosses the plasma membrane, bypasses the NPTY, and then exits at the NPVY site in the intracellular tail of LRP-1. Then, NPVY motif transmits the signal to Akt1/Akt2 kinases, which is essential to the lateral migration of keratinocytes to close the wound. Subsequent inward migration of dermal fibroblasts and dermal microvascular endothelial cells into the wound, remodels the injured tissue, and creates new blood vessels [85, 87].

In parallel, keratinocytes release extracellular vesicles containing 14-3-3σ isoforms, when the skin is damaged. This protein participates in keratinocyte-fibroblast interactions, which result in the overexpression levels of collagen and MMP1 in fibroblasts [32, 88]. Not only 14-3-3σ but also 14-3-3β and 14-3-3η isoforms seemed to induce MMP-1 expression in fibroblasts, suggesting the crucial role of 14-3-3 proteins upon epithelialization [26, 89].

Collagen, as one of the main ECM components, is effective in wound closure by attracting the keratinocytes and fibroblasts to the wound bed [90]. In association with keratinocyte EVs, EV-derived TGFβ1 is proposed to indirectly affect collagen production through the regulation of miR-21. TGF-β1 is presented to the TGFβ1RII receptor, resulting in the expression of miR-21 that targets PTEN, thereby facilitating collagen expression [52]. This regulation mechanism in wound sites is essential for controlling sustained production of collagen and subsequent attenuation of fibrosis development in pathological disorders like scars, hypertrophy, and keloids.

Relatedly, miR-21 was discovered to be related to cell migration, angiogenesis and re-epithelization. Besides, Qian et al. confirmed the potential application of miR-21-MVs derived from keratinocytes for wound healing improvement [52]. They determined that miR-21 considerably promoted skin repair in diabetic rats by stimulating fibroblast function. Stimulated fibroblast was verified to synthesize cytokines—including IL-6 and IL-8—not only to expand the wound inflammatory response but also provide a feedback loop to promote keratinocytes proliferation [91].

At present, the most noteworthy hindrance in the treatment of chronic wounds is how to prolong healing duration. Consequently, based on this evidence provided, it can be concluded that design of MVs carrying miRNA-21 might play beneficial roles, particularly in the case of diabetic ulcers.

## Conclusion

It is believed that skin-derived EVs can be considered as bioactive delivery systems that affect the function and fate of neighboring cells. For instance, keratinocyte-derived EVs contain a wide variety of biomolecules including DNA, miRNA, mRNA, and proteins that can potentially influence on the function and behavior of other skin-homing cells such as fibroblasts, melanocytes, and immune cells. It is demonstrated that the released MVs from keratinocytes can affect gene expression in fibroblasts, which may result in the proliferation, differentiation, and migration of fibroblasts or even causes fibroblast-mediated angiogenesis and pro-inflammatory responses. The physiological function of keratinocyte-derived EXs in the regulation of melanocyte proteins is also well determined, which may offer a therapeutic approach for hypo- and hyperpigmentation disorders. Furthermore, it is indicated that keratinocyte-derived EXs can function as intercellular transmitters and immune modulators through interaction with APCs, which may provide a therapeutic approach through the reduction of immune responses.

By considering these findings, it would be possible to incorporate specific therapeutic molecules like genetics materials, proteins, or even inhibitor agents into bioengineered EVs and deliver them to the target abnormal cells, i.e., fibroblasts, melanocytes, or inflammatory cells, in order to improve their biological activity for the treatment of skin disorders such as pigmentation abnormalities, autoimmune disease like psoriasis, or chronic wounds.

There is growing evidence that using EVs, or inhibitors of EVs’ components, as therapeutic cargo would be promising for treating skin disorders [9, 92]. Therefore, manufacturing of GMP-grade EVs that are then validated in preclinical and clinical studies is necessary for the translation of this technology.

## Data Availability

Not applicable.

## Abbreviations

AGO2: Argonaute 2
AMP: Antimicrobial peptide
ApoBDs: Apoptotic bodies
APC: Antigen-presenting cell
α-SMA: Alpha-smooth muscle actin
BMDC: Bone marrow-derived dendritic cells
CCN: Cellular communication network factor
CTHRC1: Collagen triple helix repeat containing 1
CXCL12: C-X-C motif chemokine ligand 12
ECM: Extracellular matrix
EGF: Epidermal growth factor
Egr-1: Early growth response 1
eEF1A1: Eukaryotic translation elongation factor 1 alpha 1
ERK1/2: Extracellular signal-regulated protein kinases 1 and 2
Emilin-3: Elastin microfibrillar interface-located protein 3
EVs: Extracellular vesicles
EXs: Exosomes
FGF: Fibroblast growth factors
HaCaT: Human adult high calcium low temperature
HBD-2: Human beta-defensin 2
hsa-miR: *Homo sapiens* microRNA
HSP70: Heat shock protein 70
HSP90α: Heat shock protein 90-alfa
HSPA5: Heat shock 70 kDa protein 5
IFNγ: Interferon gamma
ITG: Integrin
ITGA6: Integrin alpha 6
JAG1: Jagged 1
JNK: c-Jun N-terminal protein kinases
KLK7: Kallikrein 7
KRT17: Keratin 17
LDH: Lactate dehydrogenase
LFA-1: Lymphocyte function-associated antigen 1
LRP-1: Low-density lipoprotein receptor-related protein 1
MAPK: Mitogen-activated protein kinase
MMP: Matrix metalloproteinase
MHC: Major histocompatibility complex
MITF: Microphthalmia-associated transcription factor
miRNA: MicroRNA
mRNA: Messenger RNA
MAP K3: Mitogen-activated protein kinase 3
MPEK: Mouse progenitor epidermal keratinocyte
MVs: Microvesicles
NAB1: NGFI-A binding protein 1
NETs: Neutrophil extracellular traps
PAI-1: Plasminogen activator inhibitor 1
PBMCs: Peripheral blood mononuclear cells
PHKCs: Primary human keratinocyte cell cultures
PKC: Protein kinase C
PTEN: Phosphatase and tensin homolog
RECK: Reversion-inducing cysteine-rich protein with Kazal motifs
SDCBP: Syndecan binding protein
SEB: Staphylococcal enterotoxin B
TGFα: Transforming growth factor-alpha
THBS1: Thrombospondin 1
TIMP-1: Tissue inhibitor of metalloproteinase 1
TNF-a: Tumor necrosis factor-alpha
TSG101: Tumor susceptibility gene 101
TGF-β: Transforming growth factor-beta
TYR: Tyrosinase
UVB: Ultraviolet B
VEGF: Vascular endothelial growth factor
